# Sepsis and AKI: A Two-Way Street

**DOI:** 10.34067/KID.0000000000000106

**Published:** 2023-03-30

**Authors:** Abby Basalely, Shina Menon

**Affiliations:** 1 Zucker School of Medicine at Hofstra/Northwell, Division of Pediatric Nephrology, Cohen Children's Medical Center of New York, New Hyde Park, New York; 2 Department of Pediatrics, University of Washington, Division of Nephrology, Seattle Children's Hospital, Seattle, Washington

**Keywords:** acute kidney injury, pediatric nephrology

It has been well-established that sepsis is an independent risk factor of AKI; however, there is a growing body of evidence that AKI is associated with subsequent immunocompromise and risk of future infection.^1^ In 1996, Levy *et al.* studied 183 adults with contrast-related AKI and found 5.5-fold higher odds of mortality in adults with AKI compared with matched controls.^2^ Sepsis developed in 45% of patients post-AKI and was presumed to be one of the factors responsible for the higher mortality rates. Twenty years later, a secondary analysis of the Program to Improve Care in Acute Renal Disease study redemonstrated that sepsis often developed *after* AKI, seen in 243/611 (40%) critically ill adults.^3^ In this study, predictors of sepsis development post-AKI included oliguria, fluid overload, increased severity of illness scores, and dialysis requirement. Importantly, this study evaluated the temporal relationship between the two, with sepsis developing a median (interquartile range [IQR]) of 5 (2–9) days after AKI, thus establishing a time frame for surveillance and/or implementation of mitigation strategies for emerging sepsis and severe infection.

Later, studies of infection risk post-AKI suggest that it is not just the stage of AKI, severity of illness, or fluid overload that contributes to infection risk. Stage 1 AKI has been shown to confer increased odds of infection in adults undergoing nonemergent surgery as compared with patients without AKI.^4^ A review of administrative data from the Intermountain Health System found that adults who were hospitalized with AKI and had recovery of their serum creatinine to baseline by discharge had a 4.5-fold increased odds for infection within 30 days of discharge compared with peers without AKI (odds ratio 4.53 [95% confidence interval, 2.43–8.45]).^5^ The increased odds of infection in patients with a history of AKI (although attenuated) remained significantly higher in the AKI group 365 days postadmission. These data suggest that immune dysregulation continues past the acute elevation in serum creatinine and decrease in urine output, and adults with AKI may require surveillance for infection up to a year post-AKI event.

Despite the evidence in adults, the epidemiology and temporal relationship of AKI and subsequent infection in pediatric patients remains extraordinarily limited. In 2018, Soohoo *et al.* evaluated infection rates in neonates with hypoplastic left heart who underwent Norwood procedures and developed AKI. Over half of the neonates (23/42) developed a postoperative infection at a median (IQR) interval of 6 (3–13) days after AKI.^6^ In another single-center study of children with AKI on continuous kidney replacement therapy, 78% (43/55) developed an infection at a median (IQR) of 11 (4–21) days after the initiation of continuous kidney replacement therapy.^7^

In this issue of *Kidney360*, Formeck and colleagues^8^ fill this gap in knowledge by evaluating the temporal relationship of sepsis after an AKI event in 5695 children admitted to pediatric and cardiac intensive care units (ICU) at a tertiary care center between 2010 and 2014. AKI occurred in 20% (*N*=1153) of children. Although the authors planned on evaluating for sepsis over 4 weeks post-AKI event, limited data only allowed them to evaluate for 3 weeks. The incidence of hospital-acquired sepsis was twice as high in patients with AKI compared with those without (10.1% vs 4.6%), with an adjusted hazard ratio (aHR) of 1.46 (95% confidence interval 1.12–1.81). Children with severe AKI had the greatest risk (aHR 1.91) of sepsis development. The pediatric timeline for sepsis development post-AKI is similar to that reported in adults and neonates.^3,6^ Formeck *et al.*^8^ found that sepsis occurred at a median of 2.6 (1.5–4.7) days post-AKI, with 80% developing sepsis within 7 days and 97% within 2 weeks of AKI. Significant risk factors for development of sepsis post-AKI included surgery before ICU admission, male sex, higher severity of illness scores within 24 hours of ICU admission, leukopenia, and the presence of an indwelling central line. The risk of mortality was over eight times higher in those with AKI who later developed sepsis vs those who had AKI alone (5.1% vs 0.6%), highlighting the importance of infection reduction in this population.

Significantly, Formeck *et al.*^8^ point out that the risk for sepsis remained high for up to 2 weeks after AKI by which time approximately two-third of the patients had recovered kidney function on the basis of return of creatinine to baseline. These data mirror the evidence reported in adults who maintain a significant risk of infection despite the recovery of serum creatinine to baseline and further strengthen the clinical evidence of ongoing immunocompromise well past the recovery of creatinine to baseline.^5^

The underlying mechanism behind the kidney-immune system cross-talk is not very clear, and there are likely multiple pathways involved. AKI has long been considered an immunocompromised state, with critically ill patients with AKI showing evidence of impaired monocyte cytokine production and elevated plasma levels of proinflammatory cytokines.^9^ Animal models studying kidney-lung cross-talk demonstrate neutrophil dysfunction during AKI, with impaired migration and killing function.^10^ The same study found that neutrophil dysfunction may be mediated by resistin, a uremic toxin and proinflammatory cytokine, that is elevated during AKI. Recent studies of gut microbiota show that AKI can lead to dysbiosis and altered intestinal mucosal response, mediated by innate and adaptive immune responses.^11^ During AKI, there is a decrease in short-chain fatty acids, which are anti-inflammatory, and an increase in gut-derived uremic toxins which further propagate this cycle. A combination of these factors leads to the maladaptive immune response potentiating inflammation during and after AKI events.

This study had several strengths including the use of urine output criteria for AKI definition, a more sensitive marker of AKI than serum creatinine alone. The authors also accounted for immunosuppressive medication exposure in the two weeks before the AKI event. Often the factors that increase the risk of AKI are the same that increase the risk of infection. Formeck and colleagues^8^ tried to account for potential confounders, such as severity of illness, surgery, and history of malignancy, in their analysis plan using a time-varying Cox model of sepsis development. While surgery before ICU admission, severity of illness scores, leukopenia, and indwelling central lines were significant risk factors for sepsis, AKI, at any stage, remained an independent predictor of sepsis development. However, in a retrospective study like this, one can only assess association and not causality. Other limitations included a lack of information on daily fluid balance and pathologic fluid overload, an important risk factor for post-AKI sepsis in adults. The authors also note that the underlying AKI etiology was not determined; infection risk associated with AKI after cardiothoracic surgery may not be comparable with that after nephrotoxic medication–associated AKI. In addition, although the authors accounted for immunosuppressive medications before AKI, there was no information on these medications during or after the AKI event. In addition, the cohort was overwhelmingly White, and 36% were postsurgical patients, which may limit the generalizability of this study.

Although pediatric data on sepsis post-AKI are limited, the writing on the wall is clear. Patients with AKI, especially those with severe AKI, remain at increased risk of sepsis even after creatinine returns to baseline. In addition to close monitoring for sepsis, high-risk exposures, and procedures, central line placement should be limited, when clinically feasible. Prospective studies of AKI and subsequent sepsis are needed to better understand the pathophysiology of immunoparalysis and/or secondary immunocompromised state after AKI. This will help to develop targeted therapy to targets, such as proinflammatory cytokines, or modulation of the kidney-intestinal microbiome and may reduce the risk of infectious complications post-AKI.

## Disclosures

A.M. Basalely reports the following: Research Funding: Bioporto. S. Menon reports the following: Employer: University of Washington; IOV Labs (Spouse); Consultancy: Nuwellis Inc (CHF Solutions); Research Funding: Bioporto; Gerber Foundation; Honoraria: Medtronic Inc; and Advisory or Leadership Role: AKI Foundation

## Funding

None.

## References

[1] Kaddourah A Basu RK Bagshaw SM Goldstein SL. Epidemiology of acute kidney injury in critically ill children and young adults. N Engl J Med. 2017;376(1):11–20. doi:10.1056/NEJMoa1611391 27959707 PMC5322803

[2] Levy EM Viscoli CM Horwitz RI. The effect of acute renal failure on mortality. A cohort analysis. JAMA. 1996;275(19):1489–1494. doi:10.1001/jama.1996.03530430033035 8622223

[3] Mehta RL Bouchard J Soroko SB, Sepsis as a cause and consequence of acute kidney injury: program to improve care in acute renal disease. Intensive Care Med. 2011;37(2):241–248. doi:10.1007/s00134-010-2089-9 21152901 PMC3028102

[4] Griffin BR Teixeira JP Ambruso S, Stage 1 acute kidney injury is independently associated with infection following cardiac surgery. J Thorac Cardiovasc Surg. 2021;161(4):1346–1355. doi:10.1016/j.jtcvs.2019.11.004 32007252 PMC7285820

[5] Griffin BR You Z Holmen J, Incident infection following acute kidney injury with recovery to baseline creatinine: a propensity score matched analysis. PLoS One. 2019;14(6):e0217935. doi:10.1371/journal.pone.0217935 31233518 PMC6590794

[6] SooHoo M Griffin B Jovanovich A, Acute kidney injury is associated with subsequent infection in neonates after the Norwood procedure: a retrospective chart review. Pediatr Nephrol. 2018;33(7):1235–1242. doi:10.1007/s00467-018-3907-5 29508077 PMC6326095

[7] Santiago MJ Lopez-Herce J Vierge E, Infection in critically ill pediatric patients on continuous renal replacement therapy. Int J Artif Organs. 2017;40(5):224–229. doi:10.5301/ijao.5000587 28525671

[8] Formeck et al. Risk and timing of de novo sepsis in critically ill children after acute kidney injury. Kidney360. 4(3):308–315, March 2023. doi: 10.34067/KID.0005082022 36996298 PMC10103342

[9] Himmelfarb J Le P Klenzak J Freedman S McMenamin ME Ikizler TA. Impaired monocyte cytokine production in critically ill patients with acute renal failure. Kidney Int. 2004;66(6):2354–2360. doi:10.1111/j.1523-1755.2004.66023.x 15569326

[10] Singbartl K Miller L Ruiz-Velasco V Kellum JA. Reversal of acute kidney injury-induced neutrophil dysfunction: a critical role for resistin. Crit Care Med. 2016;44(7):e492–e501. doi:10.1097/ccm.0000000000001472 26646460 PMC4896858

[11] Jo SK. Kidney-gut crosstalk in AKI. Kidney360. 2021;2(5):886–889. doi:10.34067/KID.0007722020 35373056 PMC8791352

